# Menopause and Muscle: Closer to Answers, but Significant Questions Remain

**DOI:** 10.1002/jcsm.70248

**Published:** 2026-02-26

**Authors:** Stuart M. Phillips

**Affiliations:** ^1^ Department of Kinesiology McMaster University Hamilton Ontario Canada

The review by Menzies, Bowtell, Shur and Brook arrives at exactly the right moment [1]. For years, menopause has been framed, sometimes breathlessly, as a musculoskeletal ‘breaking point’ that inevitably triggers rapid skeletal muscle loss. In contrast, this review does something far more valuable. It slows the narrative down, puts human data first and makes a compelling case that our certainty about muscle loss in menopause has outrun the evidence [1]. Their central contribution is not a single new finding, but a disciplined synthesis that clarifies what the literature actually supports and what it simply has not measured.

On the question most people want answered, ‘Does menopause [the loss of estrogen and progesterone] cause muscle loss?’, the observational evidence is notably undramatic. When Menzies and colleagues focus on studies that compare menopausal stages, the average difference in DXA‐derived lean mass relative to premenopausal women is modest, roughly a few percent. They report mean differences of about −2.5% in perimenopause and −5.7% in postmenopause. That is not nothing, but it is not a cliff either [1]. Just as importantly, they highlight that many cross‐sectional comparisons necessarily include an age gap (often around a decade) between ‘pre’ and ‘post’ groups. Menopause typically (~95% of females) occurs between 45 and 55 years, meaning the transition overlaps with an entire decade of normal ageing. If ageing alone causes muscle loss at about 0.4%–0.7% per year [2], a pre‐to‐post comparison separated by about 10 years would be expected to show a roughly 4%–7% difference, even if menopause contributed nothing specific, which is not a semantic quibble. It is the difference between a claim of abrupt hormone‐driven catabolism and an interpretation more consistent with midlife ageing, behavioural (diet, activity and possibly sleep) changes and body‐composition changes operating in parallel.

There is a point at which the review crystallizes, and the review's methodological realism really helps. Menzies and colleagues are explicit that DXA does not measure skeletal muscle [1]; it measures lean body mass, a compartment that includes water, viscera and connective tissue. That matters during midlife, when body composition and hydration can shift, and when it is easy to mistake a change in ‘lean mass’ for a change in skeletal muscle contractile tissue. They point readers toward better options, including MRI [2] and creatine dilution techniques (methyl‐D_3_‐creatine) [3], which show stronger agreement with true muscle mass and may be more sensitive to change. In other words, the field has been asking a biologically specific question using tools that are often biologically non‐specific. It is unsurprising, then, that the answers have been noisy.

If muscle mass is not collapsing, could menopausal hormone therapy (MHT) prevent the small losses we see? Here, the evidence is clearer and again less exciting than popular claims. The best quantitative summary remains the systematic review and meta‐analysis by Javed and colleagues [4] (12 randomized trials, 4474 women), showing an overall difference of just 0.06 kg of lean body mass in favour of hormone therapy, which was not statistically significant (*p* = 0.26). That is a trivial signal, and it aligns with broader reviews of sex hormones and muscle adaptation. Alexander and colleagues [5] similarly conclude that any benefits of hormone therapy appear, at best, small and variable for strength, while effects on muscle mass are essentially null. If the clinical goal is meaningful preservation of skeletal muscle mass, MHT is not the lever that reliably moves it.

Where the story may shift is function, muscle quality and symptoms, which is an important nuance because women live with performance and pain, not with DXA outputs. The Baltimore Longitudinal Study of Aging analysis by Critchlow and colleagues [6] adds an intriguing layer: Across 4 to 6 years in women over 50, declines in oestradiol (and a free oestradiol index) were associated with decreases in appendicular lean mass, and a decline in free oestradiol index was associated with a decline in handgrip strength. These are observational associations, not proof of mechanism, but they are consistent with the idea that hormones may relate more closely to muscle function or to the maintenance of ‘muscle quality’ than to bulk tissue mass [6]. Even more pointedly, O'Bryan and colleagues [7] mapped neuromuscular function across the adult female lifespan and reported an accelerated decline, primarily of peripheral muscular origin, that coincided with the typical age of menopause onset. Together, these studies support a plausible reframing: Menopause may be less about sudden atrophy and more about shifts in performance, fatigability, force production or the integration of muscle with tendon and joint systems [5, 6, 7].

Mechanistically, Menzies and colleagues also highlight how uneven the human evidence remains. Some studies suggest that resting muscle protein synthesis may be elevated in older women, while anabolic responses to resistance exercise or protein ingestion may be blunted; however, direct evidence for muscle protein breakdown across the menopausal transition is notably lacking [1]. That gap matters because it is impossible to tell a hormone‐withdrawal story that hinges on increased breakdown without having the key human data to test it. Hansen [8] underscored the same theme: Female hormones plausibly influence muscle and tendon protein metabolism, but translating mechanistic plausibility into human, menopause‐specific conclusions remains challenging.

Finally, there is the clearest signal of all: symptoms. If menopause is a musculoskeletal inflection point, many women experience it as pain rather than as an abstract shift in body composition. A large systematic review and meta‐analysis reported that muscle or joint pain was present in 40% of premenopausal women, rising to 57% in perimenopause and 59% in postmenopause, with a substantially increased risk compared with premenopause and considerable heterogeneity across studies [9]. This observation is perhaps the ‘something is going on’ that deserves to be centred. Pain can reduce activity, alter movement patterns and even if lean mass changes remain modest, indirectly accelerate declines in function and muscle quality.

Menzies, Bowtell, Shur and Brook bring welcome discipline to a noisy space [1]. We have observational data that do not support a precipitous loss of lean mass during the menopausal years. We have randomized evidence showing that MHT does not meaningfully change lean mass. We also have credible signals that function, neuromuscular performance and the lived experience of musculoskeletal pain may be where menopause exerts its most important effects. The next phase is clear: better muscle measurement beyond DXA, longitudinal designs spanning late premenopause through early postmenopause, and outcomes that prioritize function and symptoms alongside tissue mass. We are closer to answers, but the questions that matter most remain open.

## Conflicts of Interest

S.P. has received grant or contract funding (paid to McMaster University) from the Canadian Institutes of Health Research, the National Science and Engineering Research Council of Canada, the US National Institutes of Health, Nestlé Health Sciences, FrieslandCampina, the US National Dairy Council, Dairy Farmers of Canada and Cargill. S.P. has received travel expenses and speaking honoraria from Nestlé Health Sciences, Optimum Nutrition, Nutricia and Danone. S.P. is an advisory board member for WndrHlth and LiquidIV. S.P. holds patents licenced to Exerkine Inc. but reports no financial gains from these patents or otherwise.

## Data Availability

Data sharing not applicable to this article as no datasets were generated or analyzed during the current study.
